# Initial Experiences of Simultaneous Laparoscopic Resection of Colorectal Cancer and Liver Metastases

**DOI:** 10.1155/2012/893956

**Published:** 2012-10-02

**Authors:** L. T. Hoekstra, O. R. C. Busch, W. A. Bemelman, T. M. van Gulik, P. J. Tanis

**Affiliations:** Department of Surgery, Academic Medical Center, University of Amsterdam, Meibergdreef 9, 1105 AZ Amsterdam, The Netherlands

## Abstract

*Introduction*. Simultaneous resection of primary colorectal carcinoma (CRC) and synchronous liver metastases (SLMs) is subject of debate with respect to morbidity in comparison to staged resection. The aim of this study was to evaluate our initial experience with this approach. *Methods*. Five patients with primary CRC and a clinical diagnosis of SLM underwent combined laparoscopic colorectal and liver surgery. Patient and tumor characteristics, operative variables, and postoperative outcomes were evaluated retrospectively. *Results*. The primary tumor was located in the colon in two patients and in the rectum in three patients. The SLM was solitary in four patients and multiple in the remaining patient. Surgical approach was total laparoscopic (2 patients) or hand-assisted laparoscopic (3 patients). The midline umbilical or transverse suprapubic incision created for the hand port and/or extraction of the specimen varied between 5 and 10 cm. Median operation time was 303 (range 151–384) minutes with a total blood loss of 700 (range 200–850) mL. Postoperative hospital stay was 5, 5, 9, 14, and 30 days. An R0 resection was achieved in all patients. *Conclusions*. From this initial single-center experience, simultaneous laparoscopic colorectal and liver resection appears to be feasible in selected patients with CRC and SLM, with satisfying short-term results.

## 1. Introduction

The liver is the most common site of hematogenous spread of primary colorectal carcinoma (CRC) [1, 2] and is affected in approximately 10–25% of patients having surgery [3]. Surgical resection is the most effective and potential curative therapy for metastatic CRC to the liver. The treatment strategies and outcomes for these patients have undergone many evolutionary changes [4–6]. Technical innovations in the field of surgery continue to evolve. Minimally invasive laparoscopic surgery improves postoperative recovery, diminishes postoperative pain, reduces wound infections, shortens hospitalization, propagates rapid return to full activity, and yields superior cosmetic results, without compromising oncological outcome [7, 8]. Currently, laparoscopic resection of primary CRC is performed in more than 40% of all patients in The Netherlands according to the Dutch Surgical Colorectal Audit [9]. However, the use of laparoscopy in liver surgery is still limited in The Netherlands [10]. 

There are several treatment options for CRC patients presenting with synchronous liver metastases (SLMs) depending on primary tumor location (rectum or colon) and extent of hepatic disease. Planning of perioperative treatment and type of surgery are discussed in a multidisciplinary team. Performing a simultaneous or staged resection of the primary tumor and liver metastasis is one of the items being discussed. Recently, a systematic review showed that combined resection resulted in shorter hospitalization and fewer complications in comparison with staged resection although a tendency was seen towards a higher postoperative mortality after simultaneous resection [11]. In contrast to the extensive literature on staged laparoscopic colorectal and laparoscopic liver surgery, there are only a few reports on combined laparoscopic colorectal and liver resection. The aim of this study is therefore to evaluate our initial experiences of simultaneous laparoscopic resection of primary CRC and SLM.

## 2. Materials and Methods

Five patients with primary CRC and a clinical diagnosis of SLM underwent combined laparoscopic colorectal and liver surgery between March 2011 and January 2012 in the Academic Medical Center, Amsterdam. Patients charts and operative notes were reviewed retrospectively for the present study. Patient and tumor characteristics, operative variables, and postoperative outcome were evaluated. 

### 2.1. Combined Modality Treatment of SLM

Treatment decisions in patients with CRC and SLM were based on location and complexity of resection of the primary tumor, extent of liver resection, feasibility of a laparoscopic approach, and physical condition of the patient. The institutional protocol of the Academic Medical Center indicates that simultaneous resection is preferred in patients who require minor liver resection (<3  segments) independent of primary tumor location. Neoadjuvant treatment preceding simultaneous resection is given for rectal primaries, consisting of short-course radiotherapy (5 × 5 Gy) followed by 3 to 6 courses of systemic chemotherapy (capecitabine and oxaliplatin). If major hepatectomy is indicated, a “liver first approach” is preferred with neoadjuvant treatment consisting of 3 to 6 courses of systemic chemotherapy which is preceded by short-course radiotherapy in case of a rectal primary. Systemic chemotherapy is completed in the adjuvant setting to a total number of 8 courses in patients with adequate clinical condition. All patients are discussed by the multidisciplinary team, and treatment is tailored to the individual patient with well-documented arguments for not following the institutional protocol (i.e., simultaneous right hemihepatectomy with right hemicolectomy in a fit young patient).

### 2.2. Surgery

**Figure 1:**
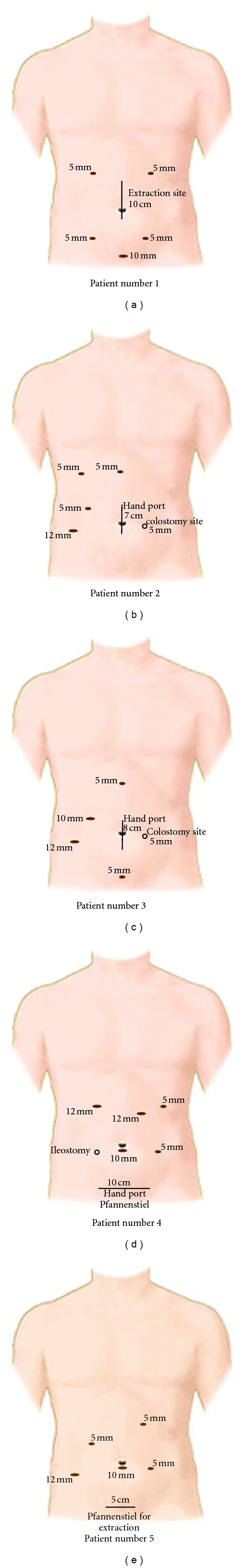
For simultaneous laparoscopic resection of colorectal cancer and liver metastases, a 10 mm subumbilical trocar was placed for pneumoperitoneum. An umbilical midline incision was created for specimen extraction in the patient that underwent a right hemicolectomy (patient number 1). In two patients, this vertical incision was used for the handport (patient numbers 2 and 3). For left-sided resections, a Pfannenstiel incision was used for specimen extraction. Four 5/12 mm trocars were positioned in the four quadrants for dissection. An extra 5 or 10 mm trocar was placed in the midline above the hand port for tumorectomy in segments 7 and 8. In one patient, an additional 5 mm trocar was placed right subcostal (patient number 2).

Laparoscopic colorectal surgery was performed using a medial to lateral approach with intracorporeal dissection and vascular control. For right hemicolectomy, a vertical umbilical incision was performed for specimen extraction and extracorporeal anastomosis. A Pfannenstiel incision was used for specimen extraction in left-sided resections followed by an intracorporeal anastomosis using the double-stapling technique with circular stapler. The specimen was extracted through the perineum after laparoscopic abdominoperineal excision. Complete laparoscopic liver resection was performed with the surgeon in between the patient's legs. For metastasectomy in segments 7 and 8, a hand-assisted laparoscopic approach was performed with the surgeon standing at the left side of the patient. Total laparoscopic liver resection was started with insertion of a 10 mm trocar at the umbilicus followed by insufflation. For tumorectomy, two 5 mm trocars were positioned in the right and left upper abdomen. For left lateral sectionectomy, two 10/12 trocars were placed in each upper quadrant and an additional 5 mm trocar left subcostally. For tumorectomy in segments 7 and 8, a hand port was placed via a vertical umbilical incision followed by a 10/12 mm trocar in the right lower quadrant and a 5 mm trocar in the midline above the hand port. In one patient, an additional 5 mm trocar was placed right subcostally. The placement of trocars is shown in Figure 1. Parenchymal transection was performed by using an ultrasonic dissection device with additional haemostasis using bipolar diathermy. The left segmental (2/3) portal pedicle and left hepatic vein were transected using a laparoscopic 60 mm stapler in case of left lateral sectionectomy. The entire liver was systematically examined to identify occult lesions using laparoscopic ultrasound. The liver specimen was put in a plastic bag in case of total laparoscopic resection and extracted via the umbilical or Pfannenstiel incision. 

## 3. Results

Four men and one woman with a pathological diagnosis of CRC and clinical or pathological diagnosis of SLM were included. The median age was 72 (range 56–77) years. Characteristics of each patient are displayed in Table 1. The average body mass index was 29.0 (range 23.9–30.1) kg/m^2^. One patient (number  5) underwent laparoscopic resection of right-sided renal cell carcinoma two months earlier. During laparoscopy, a liver lesion was found and subsequent PET scanning and endoscopy revealed an additional sigmoid tumor. Preoperative treatment of rectal cancer consisted of short-course radiotherapy (5 fractions of 5 Gy) followed by three to four courses of systemic chemotherapy (oxaliplatin and capecitabine). All laparoscopic resections were successful without conversion to open surgery. Surgical outcomes are depicted in Table 2. The following procedures were performed: total laparoscopic right hemicolectomy with tumorectomy of segment 2 (extraction via umbilical incision), total laparoscopic sigmoid resection with tumorectomy of segment 4/5 including the gallbladder and tumorectomy of segment 3 (extraction via Pfannenstiel), assisted laparoscopic low anterior resection and diverting ileostomy with total laparoscopic left lateral sectionectomy (extraction via Pfannenstiel), and two laparoscopic intersphincteric abdominoperineal excision with hand-assisted laparoscopic tumorectomy of segment 7 and segment 8, respectively (extraction via the umbilical hand port). The incision created for hand port and/or extraction of the specimen varied between 5 and 10 cm. 

Median operation time was 303 minutes (range 151–384) with an estimated total blood loss of 700 mL (range 200–850). Intraoperative complications consisted of a small perforation of the right hepatic vein during liver mobilisation in one patient, which was sutured using an additional 5 mm trocar. One patient had surgery-related complications with perineal wound infection and delayed gastric emptying (patient number 3). Other complications consisted of postoperative myocardial infarction necessitating reanimation and angioplasty (patient number 1), and pneumonia with delirium managed by intravenous antibiotics and haloperidol (patient number 2). Median postoperative hospital stay was 9 days (range 5–30). There was no postoperative mortality. An R0 resection of the primary tumor and liver lesions was achieved in all patients. Details of the pathological examination are displayed in Table 3. Definitive pathology of the liver in the patient with renal cell carcinoma and sigmoid carcinoma showed an adenocarcinoma originating from the upper gastrointestinal tract. At present, the origin of this third primary tumor is unknown and this patient receives palliative chemotherapy because of progressive liver disease. In two of the three patients who already had induction chemotherapy, systemic treatment was continued postoperatively. The patient with myocardial infarction did not receive any (neo)adjuvant systemic chemotherapy because of his poor physical condition.

## 4. Discussion

The advantages of performing laparoscopic colorectal [7] or hepatic resections [8] by experienced surgeons have led to a prominent increase for these operative approaches in recent years. Separately for primary CRC and CRC liver metastases, laparoscopic resection has been shown to result in enhanced recovery and reduced morbidity with similar oncological outcome [7, 8]. This suggests that a laparoscopic combined approach will also benefit patients who are candidates for simultaneous resection. Open resection of both primary tumor and synchronous liver metastases often requires an extensive incision, especially if the location of the liver metastasis is opposite to the primary tumor location (i.e., right liver lobe and rectum). Using a laparoscopic approach, exposure can be improved due to a magnified visualization from different angles, even in the narrow pelvis or not easily accessible places of the upper abdomen. The feasibility of a simultaneous laparoscopic approach has been demonstrated by our initial experience in five patients, and the results presented confirm findings from the limited literature on this topic (Table 4). 

Patients with a solitary, peripherally located metastasis in segments 2–6 are the most ideal candidates for simultaneous laparoscopic resection. Two additional trocars can provide adequate access to the liver besides the standard trocar placement for the colorectal procedure. Both specimens can be extracted via a single incision. Two patients in this series were diagnosed with a peripheral lesion in the posterior segments 7 and 8, requiring full mobilization of the right liver. Both liver mobilization and parenchymal transection could be accomplished in these patients using an umbilical hand port. Others also described the use of a hand port placed in an upper midline incision for liver mobilization [12, 17]. If major hepatectomy is indicated, the midline incision can subsequently be used for vascular control and parenchymal transection. But even total laparoscopic major hepatectomy in combination with colorectal resection has been shown to be feasible in three patients (Table 4) [23, 25]. This allows for a small Pfannenstiel incision to extract the specimens, which has been proven to result in the lowest incidence of incisional hernia [26].

Simultaneous resection in synchronously metastasized CRC is still controversial. There are no randomized controlled trials comparing simultaneous and staged resections and the existing comparative studies are obviously difficult to interpret due to selection bias. Theoretical arguments against simultaneous resection are the combination of a clean and contaminated procedure, and the impaired protein synthesis of the liver increasing the risk of infection and compromising anastomotic healing. Furthermore, venous congestion by Pringle maneuver may result in bowel edema. However, according to a systematic review based on 14 comparative studies, combined resection was associated with lower morbidity [11]. This led the authors to conclude that simultaneous resection can be undertaken in selected patients by surgeons specialized in both fields of colorectal and hepatobiliary surgeries. Patient selection and expertise are essential for this complex type of surgery, and the multidisciplinary team should decide on optimal timing within multimodality treatment schedules.

Life expectancy of patients with liver metastasis from colorectal origin has been increasing as a result of improvements in liver surgery and systemic chemotherapy. Repeat surgery for recurrent liver metastasis has been shown to have similar outcome compared with the first liver resection [27–29]. An initial laparoscopic approach reduces adhesion formation and facilitates repeat resection, which has also been shown to be beneficiary in patients who need subsequent liver transplantation in case of patients with HCC [30]. Given the improved oncological outcome, quality-of-life issues related to abdominal wall integrity and cosmesis become more important, underlining the potential benefits of laparoscopy.

In conclusion, our initial experience in combination with the previously published data indicate that simultaneous laparoscopic resection of primary CRC and synchronous liver metastases is feasible and advisable in selected patients, provided that appropriate expertise is available. 

## Figures and Tables

**Table 1:** Patient characteristics and preoperative data.

Patient number	Sex/age (year)	Medical history	Location CRC	Location SLM	Preoperative radiotherapy	Preoperative chemotherapy
1	M/75	Angina pectoris, paroxysmal atrial fibrillation, hypercholesterolemia, intermittent claudication, severe coronary artery disease	Ascending colon	Segment 2	No	No
2	M/77	Hypertension, appendectomy, inguinal hernia repair	Rectum	Segment 7	5 × 5 Gy	Oxaliplatin and capecitabine
3	M/72	Hypertension	Rectum	Segment 8	5 × 5 Gy	Oxaliplatin and capecitabine
4	M/56	None	Rectum	Segment 2/3	5 × 5 Gy	Oxaliplatin and capecitabine
5	V/64	Hypertension, intermittent claudication, hypothyroidism, hypercholesterolemia, resection renal cell carcinoma	Sigmoid	Segments 3, 4/5	No	No

**Table 2:** Surgical results.

Patient number	Operation	Incision (cm)/location	Operation time (min)	Blood loss (mL)	Postoperative hospital stay (d)
1	Right hemicolectomy with tumorectomy of segment 2	10/midline	151	200	30
2	Abdominoperineal excision with hand-assisted laparoscopic tumorectomy of segment 7	7/midline	310	700	9
3	Abdominoperineal excision with hand-assisted laparoscopic tumorectomy of segment 8	8/midline	384	850	14
4	Low anterior resection and diverting ileostomy with total laparoscopic left lateral sectionectomy	10/Pfannenstiel	189	800	5
5	Sigmoid resection with tumorectomy of segment 4/5 including gallbladder and tumorectomy of segment 3	5/Pfannenstiel	303	300	5

**Table 3:** Pathological examination.

Patient number	(y)pTN stage/radicality	Circumferential resection margin (mm)	Diameter SLM (cm)	Resection margin SLM (mm)
1	pT2N2/R0	—	1.5	>10
2	ypT3N0/R0	1.5	1.9	8
3	ypT0N1/R0	Not applicable (complete response)	0.6	8
4	ypT3N0/R0	>10	7.0	6
5	pT1N0/R0	—	2.7	3

**Table 4:** Case reports and small cohort series describing laparoscopic colorectal surgery in combination with liver surgery using different approaches. Indication was colorectal cancer except for Inagaki et al. [12] (diverticular disease with cystic liver tumor).

Author	Year	*N*	Liver resection		Time (min)	Blood loss (mL)	LOS (days)
			Laparoscopic assisted	Total laparoscopic			
Inagaki et al. [12]	2003	1	1 LH	0	331	930	16
Geiger et al. [13]	2006	1	0	1 LLS	330	600	4
Leung et al. [14]	2006	1	0	1 LLS	350	500	7
Vibert et al. [15]	2006	8	0	8	NR	NR	NR
Law et al. [16]	2008	4	0	4	NR	NR	NR
Kim et al. [17]	2008	3	2 S	0	362	300	10
			1 T		(210–450)	(300-300)	(9–16)
Pessaux and Panaro [18]	2009	1	0	1T + RFA	NR	NR	NR
Bretagnol et al. [19]	2008	3	0	1 LLS	NR	NR	NR
				2 T			
Sasaki et al. [20]	2009	9	0	2 LLS	418	219	9
				7 T	(215–520)	(32–745)	(7–26)
Akiyoshi et al. [21]	2009	3	3 T	0	372	45	16
					(300–453)	(30–60)	(16–23)
Casaccia et al. [22]	2010	1	0	1 LLS	455	NR	12
Lee et al. [23]	2010	10*	0	6 LLS	401	500	10
				5 T	(230–620)	(60–1000)	(7–15)
				1 S			
				1 RH			
Hayashi et al. [24]	2011	4	2	2	378	138	11
					(270–575)	(40–330)	(7–14)
Tranchart et al. [25]	2011	2	0	1 LH	310, 345	200, 200	4, 6
				1 RH			

LOS: length of postoperative hospital stay, NR: not reported, LH: left hemihepatectomy, LLS: left lateral sectionectomy, T: tumorectomy, S: segmentectomy, RH: right hemihepatectomy, RFA: radio frequency ablation, *13 resections in 10 patients.
